# Sacral neuromodulation remote programming in patients with refractory lower urinary tract dysfunction: China’s experience during the COVID-19 pandemic

**DOI:** 10.3389/fmed.2023.977433

**Published:** 2023-03-13

**Authors:** Lingfeng Meng, Huimin Hou, Peng Zhang, Yinjun Gu, Benkang Shi, Yan Li, Qingwei Wang, Yifei Zhang, Lijuan Ren, Qi Chen, Zhen Yuan, Fan Guo, Dianyou Li, Yunfu Ma, Sheng Dong, Zhijun Liu, Aijia Shang, Bo Li, Wei Xu, Jianwei Lv, Yaoguang Zhang

**Affiliations:** ^1^Department of Urology, Beijing Hospital, National Center of Gerontology, Institute of Geriatric Medicine, Chinese Academy of Medical Sciences, Beijing, China; ^2^Department of Urology, Beijing Hospital Continence Center, Beijing, China; ^3^Department of Urology, Beijing Chaoyang Hospital, Institute of Urology, Capital Medical University, Beijing, China; ^4^Department of Urology, Renji Hospital Affiliated to Shanghai Jiaotong University, Shanghai, China; ^5^Department of Urology, Qilu Hospital of Shandong University, Jinan, China; ^6^Department of Urology, The First Affiliated Hospital of Zhengzhou University, Zhengzhou, China; ^7^Department of Urology, The First Affiliated Hospital of Anhui Medical University, Hefei, China; ^8^Department of Urology, The First Hospital of Shanxi Medical University, Taiyuan, China; ^9^Department of Urology, The Second Affiliated Hospital of Xi’an Jiaotong University, Xi’an, China; ^10^Department of Urology, Fuyang People's Hospital, Fuyang, China; ^11^Department of Urology, Hubei Provincial Hospital of TCM, Wuhan, China; ^12^Department of Neurosurgery, Ruijin Hospital, Medical College, Shanghai Jiaotong University, Shanghai, China; ^13^Department of Neurosurgery, The Third Affiliated Hospital of Zhengzhou University, Zhengzhou, China; ^14^Department of Neurosurgery, Beijing Tsinghua Changgung Hospital, Beijing, China; ^15^Department of Urology, Qingdao Municipal Hospital, Qingdao, China; ^16^Department of Neurosurgery, Chinese PLA General Hospital, Beijing, China; ^17^Department of Urology, Shandong Provincial Hospital, Jinan, China; ^18^Department of Urology, Zoucheng People’s Hospital, Zoucheng, China; ^19^Department of Urology, Shanghai Pudong New Area Gongli Hospital, Shanghai, China

**Keywords:** lower urinary tract dysfunction, sacral neuromodulation, remote programming, COVID-19, urinary bladder

## Abstract

**Objectives:**

Sacral neuromodulation is an effective, minimally invasive treatment for refractory lower urinary tract dysfunction. However, regular postoperative programming is crucial for the maintenance of the curative effects of electronic sacral stimulator devices. The outbreak of coronavirus disease 2019 (COVID-19) limited the ability of practitioners to perform traditional face-to-face programming of these stimulators. Therefore, this study aimed to evaluate the application of remote programming technology for sacral neuromodulation during the COVID-19 pandemic in China.

**Materials and methods:**

We retrospectively collected data including baseline and programming information of all patients with lower urinary tract dysfunction who underwent sacral neuromodulation remote programming in China after the outbreak of COVID-19 (i.e., December 2019). The patients also completed a self-designed telephone questionnaire on the subject.

**Results:**

A total of 51 patients from 16 centers were included. They underwent 180 total remote programming visits, and 118, 2, 25, and 54 voltage, current, pulse width, and frequency adjustments, respectively, were performed. Additionally, remote switching on and off was performed 8 times; impedance test, 54 times; and stimulation contact replacement, 25 times. The demand for remote programming was the highest during the first 6 months of sacral neuromodulation (average, 2.39 times per person). In total, 36 out of the 51 patients completed the questionnaire survey. Of these, all indicated that they chose remote programming to minimize unnecessary travel because they had been affected by COVID-19. The questionnaire also showed that remote programming could reduce the number of patient visits to the hospital, save time, reduce financial costs, and would be easy for patients to master. All surveyed patients indicated that they were satisfied with remote programming and were willing to recommend it to other patients.

**Conclusion:**

Remote programming for sacral neuromodulation is feasible, effective, safe, and highly recommended by patients with refractory lower urinary tract dysfunction. Remote programming technology has great development and application potential in the post-pandemic era.

## 1. Introduction

Lower urinary tract dysfunction (LUTD) refers to the dysfunction of the bladder and urethra during urinary storage and/or ejection periods, which often show corresponding symptoms (1). LUTD could be caused by various diseases, including overactive bladder, neurogenic bladder, interstitial cystitis/bladder pain syndrome, and non-obstructive urinary retention. Its pathogenesis is complex, and traditional treatment often cannot achieve satisfactory outcomes.

Sacral neuromodulation (SNM) has been used in the treatment of refractory LUTD for over 30 years and was approved for clinical use by the Food and Drug Administration of the United States in 1997 (2, 3). In China, SNM has been used for nearly 10 years and has been further developed and promoted in recent years (4–6). Although SNM can effectively improve the clinical symptoms of refractory LUTD, the degree of symptom improvement may gradually decrease with disease progression. Therefore, to maintain a satisfactory curative effect, patients often need to visit the hospital regularly for programming their neuromodulator devices.

Currently, most medical centers qualified to perform SNM in China are located in larger cities, even though China has a vast territory with uneven regional medical development, making it difficult to adjust and construct follow-up plans for many patients after medical treatment. These patients often spend more time and incur larger economic costs visiting qualified medical centers for programming, which affects patient enthusiasm and medical compliance, resulting in poor long-term postoperative outcomes.

To address this, the Tsinghua PINS company developed a new SNM product and adopted a unique remote programming technology that allows patients to contact their doctors from anywhere with internet connectivity to adjust parameters and stimulation parameters. This technology has enabled patients to avoid many of the aforementioned difficulties—especially since the start of the coronavirus disease 2019 (COVID-19) pandemic in December 2019. Many countries, including China, have imposed strict quarantine measures due to the high transmissibility of severe acute respiratory syndrome coronavirus 2 (SARS-CoV-2). During such quarantine periods in China, travel was highly restricted. Therefore, patients were unable or could only rarely visit qualified hospitals for programming, even if their symptoms fluctuated, which led to a surge in patient demand for remote programming options.

Considering that COVID-19 is likely to become a seasonal disease in the foreseeable future, we analyzed the data and feedback of all patients who received SNM remote programming in China since the pandemic began. This study aimed to evaluate the application of SNM remote programming in patients with LUTD during the COVID-19 pandemic in China and further improve the technology so as to better provide services for patients.

## 2. Materials and methods

### 2.1. Clinical data

Data including medical records, stimulus parameters, and adverse events of all patients with refractory LUTD in Chinese medical centers practicing SNM remote programming were retrospectively collected and analyzed. The inclusion criteria were patients who (1) underwent SNM implantation (PINS, Beijing, China) for refractory LUTD, (2) underwent remote programming from December 2019 to April 2021, (3) were compliant and could cooperate to complete the follow-up, (4) aged ≥14 years, and (5) provided informed consent. The exclusion criteria were (1) severe mental or cognitive impairment or inability to respond correctly during programming, (2) comorbidities that affect life expectancy, (3) poor general patient condition, and (4) any situation that the researchers considered inappropriate.

### 2.2. Self-designed questionnaire

By searching the relevant literature and comparing previous findings to our study population, we designed a questionnaire and conducted a telephone questionnaire for all the participating patients. The contents of the questionnaire included the time and economic costs (including accompanying family members) of conventional programming each time the patient visited the hospital clinic, the reason for applying for remote programming, the expected frequency of remote programming, the perceived benefit received from remote programming with regard to symptoms, degree of difficulty implementing remote programming, overall patient satisfaction with remote programming, and likelihood to recommend the process to others. Informed consent was obtained from all participants, and all clinical data were collected only after approval of the study protocol by the Ethics Department of Beijing Hospital (Approval no: 2020BJYYEC-141-01), whose institutional guidelines were followed during the study.

### 2.3. Introduction, implementation, and security of the remote programming system

The use of the remote programming system in deep brain stimulation (DBS) was first reported in 2015, and we believe this method has been feasible and effective for postoperative follow-ups in DBS (7). Since then, Tsinghua PINS Company has developed and launched a new remote programming system in 2019 and applied it to DBS, spinal cord stimulation (SCS), and SNM (8–10).

**Figure 1:**
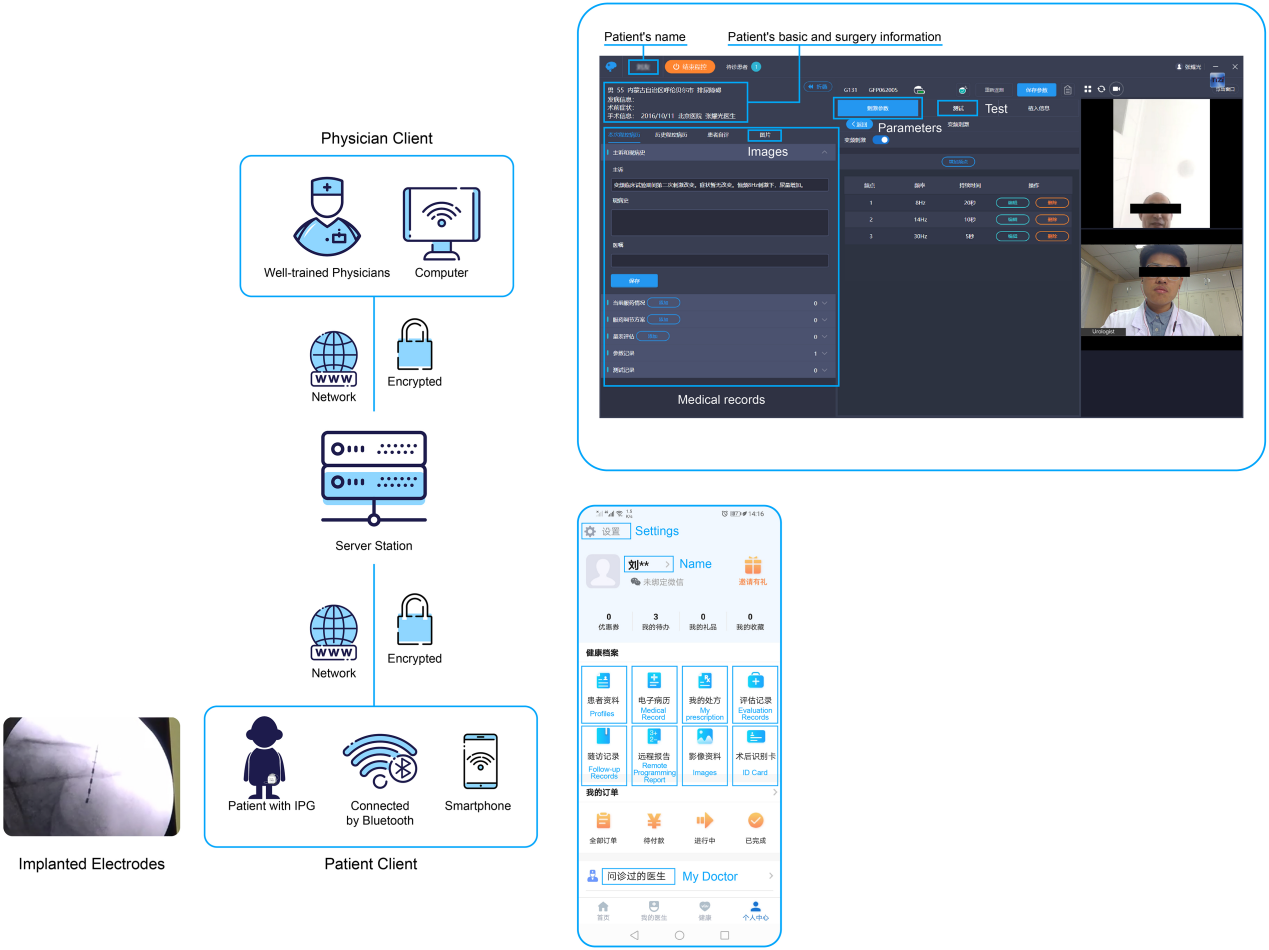
Overall architecture of the remote programming system. The remote programming system consists of three parts (“sides”): the doctor, patient, and server sides. The patient and doctor sides are connected with the server side through two different versions of the application. The upper and lower right panels show the doctor and patient versions, respectively, of the application.

This remote programming system consists of three interface “sides”: the doctor, the patient, and the server side. The patient and doctor sides are connected to the server side through two versions of the application (Figure 1). Specifically, the patient connects to a Wi-Fi or 4G/5G network through a mobile phone at home, logs in to the patient version of the app, and selects and books an appointment with a doctor. When performing remote programming, the patient first needs to match the implanted pulse generator through Bluetooth. After the match is successful, the patient’s video, voice, and stimulation signals can be transmitted through the network to the PINS Remote Tech center (i.e., the server side).

The doctor logs in to the doctor’s version of the client, where they can adjust the neuroregulatory treatment program accepted by the patient according to the patient’s condition. The principle is that the doctor sends programming instructions to the server side through the client, and the server side converts the instructions into operation instructions that can be recognized by the stimulator, then sends these instructions to the patient client to adjust functions such as audio/video communication, telemetry, parameter adjustment, electrode impedance detection, and others. Chinese scholars have proven that this technology is not inferior to face-to-face programming (11, 12).

We implemented three-level measures to ensure the security and confidentiality of all data in the transmission process. The first level of protection was the use of the Hypertext Transfer Protocol (HTTP) over a secure socket layer in the doctor, patient, and server data transmissions, and the use of asymmetrical encryption two-way authentication technology. The second protection measure was the use of a secure low-power Bluetooth module with identity authentication encryption. The third protective measure was the improvement of equipment communication in the near-field communication mode, which prevented the possibility of misconnection and unintended activation. In addition, the patient version of the application also has a “one-click recovery/close” function to prevent unexpected outcomes caused by network problems during remote programming.

## 3. Results

A total of 51 patients from 16 centers were enrolled in this study: 22 men (43.1%) and 29 women (56.9%). The average patient age and follow-up time from SNM implantation to the last follow-up were 45.88 ± 20.64 years and 10.16 ± 6.87 months, respectively. The clinical diagnostic classification and other baseline information of the patients are shown in Table 1.

**Table 1:** Baseline information.

Patient baseline information	(*N* = 51)
Age (years)	45.88 ± 20.64
Follow-up time (months)	10.16 ± 6.87
Sex	
Male	22 (43.1)
Female	29 (56.9)
Clinical diagnostic classification	
Interstitial cystitis/painful bladder syndrome	8 (15.7)
Overactive bladder	6 (11.8)
Dysuria	5 (9.8)
Neurogenic bladder	32 (62.7)
Implantation site	
Left side	22 (43.1)
Right side	27 (53.0)
Bilateral	2 (3.9)
Residence	
Urban	35 (68.6)
Rural	16 (31.4)

Values are expressed as mean ± standard deviation and *n* (%).

During the COVID-19 pandemic, 51 patients underwent 180 remote programming sessions, and 118, 2, 25, and 54 voltage, current, pulse width, and frequency adjustments, respectively, were performed. The frequency parameter had the largest adjustment range, up to 1,686%. Remote switching between on and off states was performed eight times, impedance testing was performed 54 times, and stimulation contact replacement was performed 25 times. Stimulation mode was changed once, from “cycle mode” (regular switching between on/off states) to “continuous mode.”

In addition to the specific remote programming parameter adjustments, we also measured the frequency of patient demand for remote programming in different postoperative periods. Similar to traditional face-to-face programming, the greatest demand for remote programming was within 6 months postoperatively (average, 2.39 times per person). After that, the frequency of remote programming decreased significantly, and the average frequencies from 6 months to 1 year and after 1 year postoperatively were 0.98 and 0.16 times per person, respectively.

**Figure 2:**
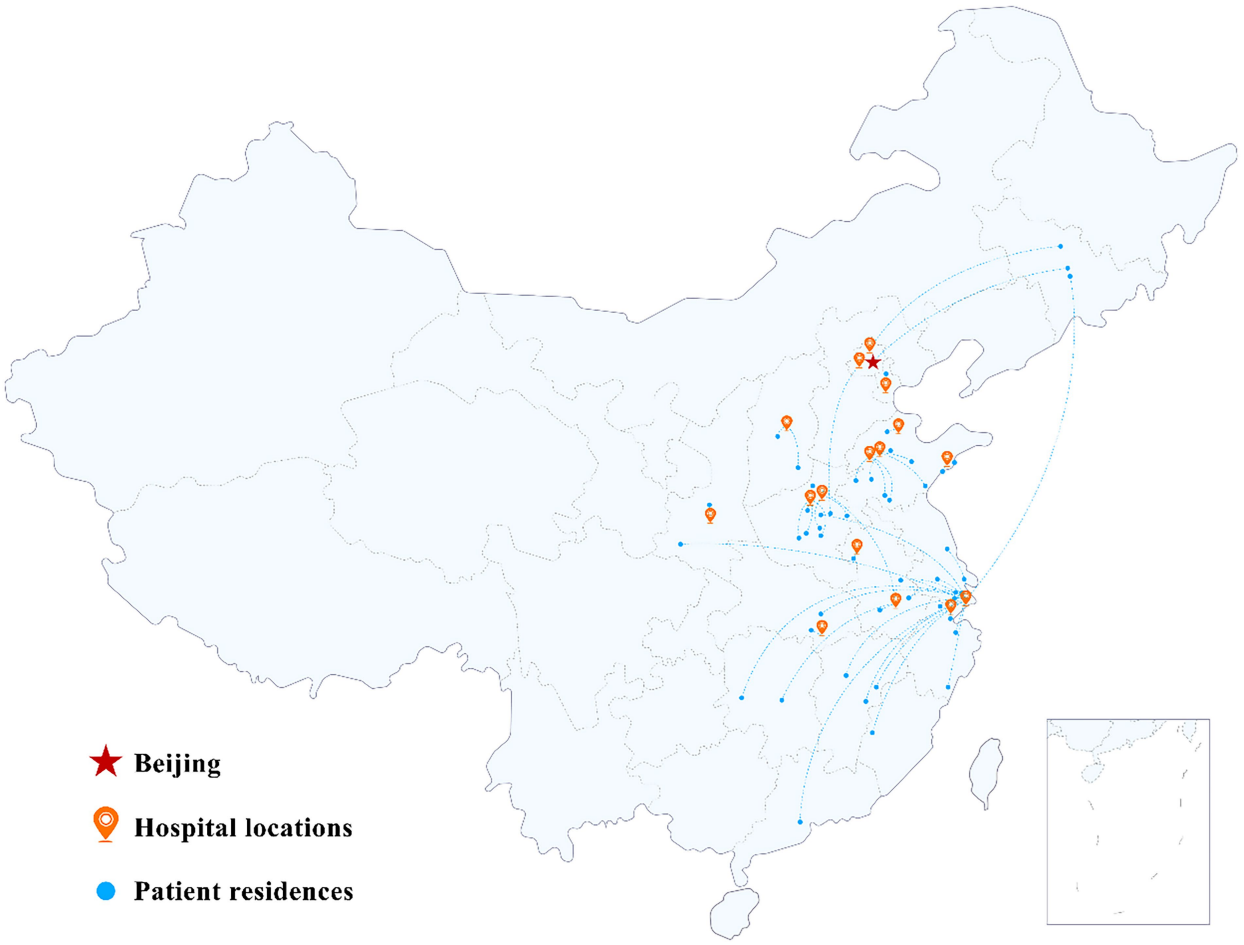
Map of the patient residences and hospital locations. The red star, orange location symbols, and blue dots represent Beijing, hospital locations, and patient residences, respectively.

Patient residences and hospital locations are shown in Figure 2. One patient, whose residence is not shown in the figure, settled in Canada. It is worth noting that 68.6% of the patients included lived in urban areas, and the average home–hospital distance was approximately 400 km (range, 2–2,161 km).

The questionnaire results showed that the economic cost of patient visits to the hospital outpatient clinic was relatively low, and nearly half of the patients incurred a cost of <500 yuan (Table 2). However, the time cost was relatively high, with 70% of patients spending more than a day in the hospital during these visits.

**Table 2:** Questionnaire results.

Questionnaire survey result	(*N* = 36)
Cost of face-to-face regular programming (yuan)	
<500	17 (47.2)
500–2,000	13 (36.1)
2,000–5,000	1 (2.8)
5,000–10,000	5 (13.9)
Time-consumption of face-to-face regular programming (hours)	
<12	10 (27.8)
12–24	12 (33.3)
24–48	10 (27.8)
≥48	4 (11.1)
Reasons for applying for remote programming (multiple choices)	
Recurrent symptoms	13 (36.1)
For further improvement	21 (58.3)
Discomfort in the current stimulus parameters	0
Regular follow-up	12 (33.3)
Expected frequency of remote programming	
Once a week	1 (2.8)
Once every 1–3 months	5 (13.9)
Once every 3–6 months	4 (11.1)
Once a year	1 (2.8)
When needed	25 (69.4)
Reasons for choosing remote programming (multiple choices)	
Professional, can achieve expert-level service	17 (47.2)
Timely programming adjustment can be obtained	13 (36.1)
Save time and economic cost	32 (88.9)
Improve the curative effect	11 (30.6)
Affected by COVID-19, travel should be reduced as much as possible	36 (100)
Question: Can you understand the operation process and use the remote programming platform?	
Yes.	29 (80.6)
Still unable to understand and use under the guidance of others	7 (19.4)
Satisfaction	
Very satisfied	20 (55.6)
Satisfied	13 (36.1)
Basically satisfied	3 (8.3)
Not satisfied	0
Recommended level	
Excellent	15 (41.7)
Good	16 (44.4)
Fair	5 (13.9)
Poor	0

Values are expressed as *n* (%).

Most patients applied for remote programming due to recurrent symptoms, hoping for further improvement. Compared with traditional face-to-face programming, the new remote programming system reduces time, travel, and economic costs to patients as much as possible, which is significant during the current COVID-19 pandemic. There was a good acceptance of the remote programming system, and 80.6% of patients indicated that they were able to understand and use the system under the guidance of technicians or doctors. All patients had a satisfactory experience and were willing to recommend remote programming to others. No adverse events were observed in any of the cases.

## 4. Discussion

Since SNM was first used by Tanagho in 1981 to treat bladder dysfunction, it has been continuously improved upon and an increasing number of patients have received SNM treatment.^2^ After 40 years of development, SNM has become one of the most important minimally invasive treatments for refractory LUTD. However, with the gradual increase in implantation operations and the number of medical centers conducting this type of operation in China in recent years, some patients have experienced reduced efficacy or even failure of the follow-up process (13). A 14-year follow-up study by Al-zahrani et al. showed that approximately 23% of patients received a second surgical intervention after SNM due to the poor efficacy of the treatment (14). However, Chinese experts have a conservative attitude toward secondary surgical intervention, believing that it must be conducted only in cases where combined oral medications and repeated adjustments of stimulation parameters have proven ineffective (15, 16). A recent meta-analysis described the efficacy of different combinations of stimulus parameters on LUTD, affirming the importance of programming to ensure the efficacy of SNM (17). Therefore, regular programming is an important means of ensuring a stable curative effect and reducing the probability of refractory patients with LUTD requiring second surgical interventions.

Similar to other neuromodulation therapies such as SCS and DBS, postoperative stimulator programming for SNM often requires several attempts to find the appropriate parameter combination for each patient. This process is generally completed within 6 months postoperatively, placing a heavy burden on the patient. This study showed that the patient demand for remote programming was the highest within the first 6 months postoperatively, after which it decreased significantly. A similar conclusion has been reached for DBS (18, 19). As an increasing number of China’s more remote provincial hospitals gradually begin to perform SNM surgeries, patients no longer have to travel to larger cities for surgery and programming. Therefore, the economic cost of patients visiting the hospital for traditional face-to-face programming is lower than before. However, more than 70% of the patients in our study needed to spend a full day or more in the hospital for programming visits, likely because Chinese doctors in large medical centers (at or above the provincial level) are often overburdened, which leads to increases in the time cost of the patient consultation process.

Remote programming for SNM devices has recently been developed, and its implementation for patients with SNM began in 2019. Its safety and effectiveness have since been thoroughly confirmed (11, 12). China reported its first case of SARS-CoV-2 infection in December 2019, and it was declared a pandemic by the World Health Organization in 2020 (20). COVID-19 then spread rapidly worldwide due to international travel, resulting in outbreaks in more than 200 countries. Since the outbreak of COVID-19, the number of infections and deaths continued to increase. As of November 2020, there have been more than 50 million confirmed cases and more than 1 million deaths worldwide (21). As the first country to take measures to prevent and control the epidemic, China began to provide telemedicine services as early as February 2020, aiming to alleviate citizens’ anxiety and reduce unnecessary travel (22). In June of the same year, the United States also abolished provisions restricting the provision of telemedicine services to rural areas, allowing for the reimbursement of telemedicine services for everyone with medical insurance (23). In addition, China has been at the forefront of citizen education. Perhaps in light of these conditions, all patients in this study indicated that the most important reason for choosing remote programming was to help them comply with governmental rules and recommendations by minimizing travel during the COVID-19 pandemic. This proportion of patients exceeded that of those who chose the method solely to save time and economic costs.

Considering that the remote programming system operates using electronic equipment, this study explored whether it posed additional operative difficulties for patients. However, the acceptability of remote programming was positively evaluated by our patients, with over 80% indicating that they were able to master remote programming and use it proficiently. The patients in this study were relatively young, and it is believed that, after adequate explanation and training, the proportion of young patients with SNM using this method will continue to increase. All the patients in our study affirmed the necessity and convenience of remote SNM programming and expressed satisfaction with the technology and willingness to recommend it to others. Overall, our survey confirmed that remote programming was satisfactory and effective. A single remote programming session saves at least 500 yuan for nearly half of the patients on average and saves at least 12 h of time for 70% of patients. Remote programming can reduce the number of hospital visits needed by patients, time, and economic costs to patients and the healthcare system, and is easy to operate in a way that allows patients to quickly master the process.

In addition, remote programming eliminates the inevitable hassle of traditional face-to-face programming so that patients have enough energy to cooperate with doctors to complete necessary medical history collection and other work. Compared with the noisy environments commonly seen in hospital outpatient clinics and the possible risk of infectious disease transmission at these facilities, doctors and patients can communicate in a quieter and safer environment through remote programming. On the contrary, remote programming is also in high demand among doctors. For the surgeon, most immediate feedback is received through postoperative programming. Through the remote programming platform, doctors can make more timely responses to the programming needs of patients, in a way that is more convenient for continuous postoperative management of outpatients.

The main strength of this study is that it provides important insights regarding the management of patients with SNM during the COVID-19 pandemic. Through our systematic statistical analysis of the data of all the patients who received remote programming during the COVID-19 pandemic in China, as well as the results of our questionnaire survey, first-hand information was obtained. This information can provide a reference for further medical development in the post-pandemic era. On the contrary, this study has some key limitations as well. First, the sample size was small. Due to the relatively high cost of SNM, it is still a treatment that is only available to a segment of the population in China. However, SNM was included in the Beijing Medical Insurance List in 2020. It is, therefore, believed that an increasing number of patients will receive SNM surgery in the future. Second, this study does not compare remote programming with traditional programming and cannot explain the advantages and disadvantages of both. Third, this study was based on the subjective satisfaction of patients and did not objectively evaluate the effectiveness of remote programming for symptom improvement. Fourth, it is unclear whether the attitudes of patients toward remote programming were affected by COVID-19. These limitations need to be explored further.

## 5. Conclusion

This study confirms that remote programming has great development and application potential in the post-pandemic era. Through clinical implementation and patient feedback, we determined that the success rate, effectiveness, safety, and patient satisfaction with this method were all high. With further development and the popularization of remote programming technology, it is believed that remote SNM programming can provide more efficient, economical, and convenient program-controlled services for patients with LUTD.

## Data availability statement

The raw data supporting the conclusions of this article will be made available by the authors, without undue reservation.

## Ethics statement

All clinical data were collected only after the approval of the study protocol by the Ethics Department of Beijing Hospital (Approval no: 2020BJYYEC-141-01). The patients/participants provided their written informed consent to participate in this study. Written informed consent was obtained from the individual(s) for the publication of any potentially identifiable images or data included in this article.

## Author contributions

LM and YaZ: conception and design. YaZ: administrative support. LM, HH, and PZ: collection and assembly of data. LM and JL: data analysis and interpretation. LM, HH, PZ, YG, BS, YL, QW, YiZ, LR, QC, ZY, FG, DL, YM, SD, ZL, AS, BL, WX, JL, and YaZ: provision of study materials or patients, manuscript writing, and final approval of manuscript.

## Funding

This study was funded by the National Key Research and Development Program of China (2018YFC2002202) and the National High Level Hospital Clinical Research Funding (BJ-2021-184).

## Conflict of interest

The authors declare that the study was conducted in the absence of any commercial or financial relationships that could be construed as a potential conflict of interest.

## Publisher’s note

All claims expressed in this article are solely those of the authors and do not necessarily represent those of their affiliated organizations, or those of the publisher, the editors and the reviewers. Any product that may be evaluated in this article, or claim that may be made by its manufacturer, is not guaranteed or endorsed by the publisher.

## Supplementary material

The Supplementary material for this article can be found online at: https://www.frontiersin.org/articles/10.3389/fmed.2023.977433/full#supplementary-material
